# Promiscuity, a Driver of Plant Cytochrome P450 Evolution?

**DOI:** 10.3390/biom13020394

**Published:** 2023-02-18

**Authors:** Danièle Werck-Reichhart

**Affiliations:** Institut de Biologie Moléculaire des Plantes du Centre National de la Recherche Scientifique (CNRS), Université de Strasbourg, 67000 Strasbourg, France; werck@unistra.fr; Tel.: +33-6-13549086

**Keywords:** cytochrome P450, plant metabolism, promiscuity, evolution, plant defense, herbicide resistance

## Abstract

Plant cytochrome P450 monooxygenases were long considered to be highly substrate-specific, regioselective and stereoselective enzymes, in this respect differing from their animal counterparts. The functional data that have recently accumulated clearly counter this initial dogma. Highly promiscuous P450 enzymes have now been reported, mainly in terpenoid pathways with functions in plant adaptation, but also some very versatile xenobiotic/herbicide metabolizers. An overlap and predictable interference between endogenous and herbicide metabolism are starting to emerge. Both substrate preference and permissiveness vary between plant P450 families, with high promiscuity seemingly favoring retention of gene duplicates and evolutionary blooms. Yet significant promiscuity can also be observed in the families under high negative selection and with essential functions, usually enhanced after gene duplication. The strategies so far implemented, to systematically explore P450 catalytic capacity, are described and discussed.

## 1. Introduction

Cytochrome P450s (CYPs) were first detected in mammalian livers and it soon appeared that a small subset of only five CYP enzymes highly expressed in the liver, out of 57 encoded by the human genome, were responsible for the metabolic clearance of 90% of ingested drugs [1]. This led early-on to distinguish two groups of mammalian CYPs: one catalytically promiscuous group thought to have evolved for detoxication of exogenous chemicals like food plant-sourced compounds, and the other group with more restricted substrate specificity and with essential roles in the homeostasis of endocrine signaling compounds such as steroid hormones, oxylipins, vitamin D or retinoids. Yet, this functional division that initially appeared clear-cut became blurred as the field developed, showing that CYPs involved in the signaling metabolism also sometimes contributed to the conversion of xenobiotics [2].

Conversely in plants, CYPs were initially regarded as catalytically selective and highly specialized enzymes, controlling hormone homeostasis, the biosynthesis of structural building blocks, and the formation of a broad range of taxon-specific specialized metabolites mediating fine-tuned defense or cross-talk with other organisms. This was due to the properties of the first enzymes that were thoroughly investigated, showing high substrate-, regio- or stereo-specificity, and to the large and divergent CYP gene complement present in each plant genome. Nevertheless, as early as the 1960s, plant CYPs were reported to metabolize herbicides [3,4] and recently, the work of several groups have converged to demonstrate the role of a few members of the CYP81 family in the metabolism of a quite broad range of herbicides in grasses [5,6]. This has fed speculations on the evolution of a group similar to the drug-metabolizing mammalian CYP in plants, having specialized for detoxication of exogenous chemicals. Yet, as expected for a highly diversified superfamily of enzymes, especially for the plant CYP families showing frequent gene blooms (i.e., taxon-specific gene family expansions), relaxed substrate specificity is more commonly encountered than initially thought.

Until recently, the catalytic permissiveness of the plant CYP enzymes was only superficially documented. This is because most investigators test and evaluate enzyme activities in the context of metabolic pathways, based on a prior hypothesis that they expect to confirm. So any sign of even minimal activity is accepted as a proof that the physiological and hence best substrate was identified. This might not always be the case, especially when the substrate investigated is small, very hydrophobic and/or flexible as was already noted for fatty acids in the case of mammalian enzymes [2].

In the last decade, the catalytic permissiveness of plant CYPs with important developmental or ecological functions has been more thoroughly investigated in some cases. This work, together with serendipitous discoveries, disclosed unexpected substrate promiscuity and its result on plant metabolic complexity, development and adaptation. In this review, I summarize some of the data revealing plant CYP catalytic and functional plasticity. I also discuss the potential role of this plasticity in the evolution of plant metabolism and its possible applications. My main focus is the capacity to convert a multiplicity of substrates (substrate promiscuity) or to catalyze different reactions or oxidations at different sites on the same substrate (catalytic promiscuity) rather than multifunctionality (capacity to perform a cascade of oxidations on the same compound) or promiscuity of products, both of which are also commonly observed with plant CYP enzymes.

## 2. The Diterpenoid Metabolism

**Figure 1:**
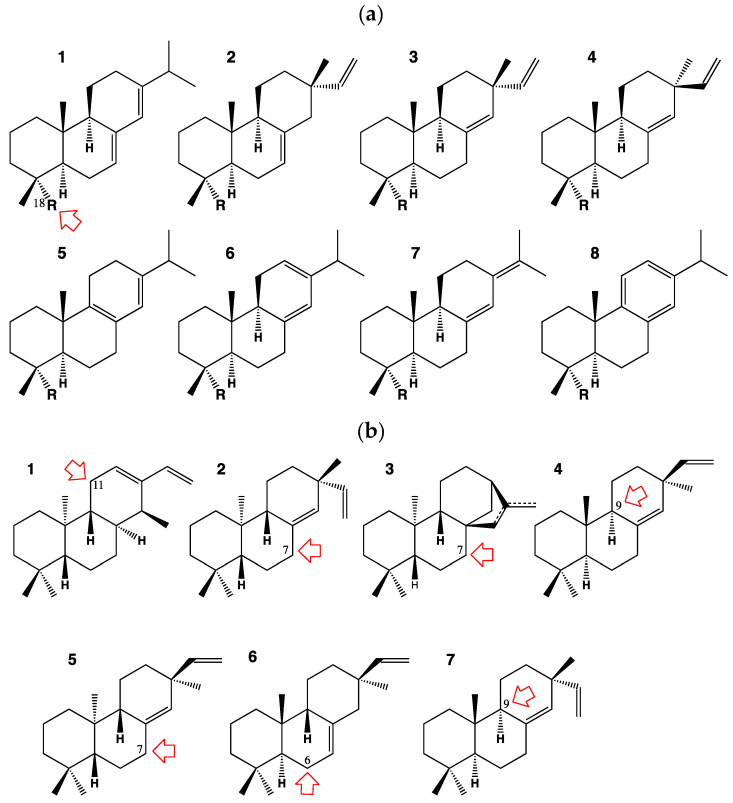
First examples of CYP promiscuity reported in the diterpenoid metabolism: (**a**) Diterpenoid substrates of CYP720B4 of sitka spruce. All compounds are attacked on C18 whether as methyl, alcohol, aldehyde substituent: **1:** abietadiene; **2**: isopimaradiene; **3**: sandaracopimaradiene; **4**: pimaradiene; **5**: palustradiene; **6**: levopimaradiene; **7:** neoabietadiene; **8**: dehydroabietadiene. (**b**) Diterpenes susbstrates of CYP76M8 of rice. Position of oxidative attack on each compound is numbered and indicated by the red arrow. Via these reactions CYP76M8 contributes to the phytocassane, oryzalexin and momilactone pathways: **1**: *ent*-cassa-12,15-diene; **2**: *ent*-pimara-8(14),15-diene; **3**: *ent*-(iso)kaurene; **4**: pimara-8(14),15-diene; **5**: *ent*-sandaracopimaradiene; **6**: *syn*-pimara-7,15-diene; **7**: sandaracopimaradiene.

The first well-supported evidence of plant CYP promiscuity arose from analyses of diterpenoid metabolism in monocots and conifers. Such taxa usually form arrays of structurally-related compounds via initial action of multiple terpene synthases on geranylgeranyl diphosphate. In conifer trees of the pine family, diterpene acids are major constituents of the oleoresin defense. The CYP720B subfamily is conifer-specific. It was found to be active in the biosynthesis of the diterpenoid constituents belonging to different structural groups in conifer oleoresins. One of its members, CYP720B1 from the loblolly pine, was first shown to oxidize the alcohol and aldehyde derivatives of four closely related diterpene isomers [7]. Later on, via expression in yeast/*E. coli* and RNAi suppression in white spruce, it was demonstrated that CYP720B4 from the Sitka spruce catalyzes the three successive oxidations at C18 of 8 out of 24 different diterpenoid olefin skeletons to form the corresponding alcohols, aldehydes and acids [8] (Figure 1a).

Monocot defense is also largely supported by a diversified set of diterpene phytoalexins. In rice, it was found that members of the CYP76M subfamily are active on several diterpene structures, with the most promiscuous of them, CYP76M8, catalyzing hydroxylation of seven different diterpene skeletons at different positions and thus potentially contributing to the formation of three different rice phytoalexin pathways in rice (phytocassanes, oryzalexins and momilactones) [9] (Figure 1b). Catalytic permissiveness was also observed for other families contributing to these pathways, such as CYP701, CYP99 or CYP71, although to a lesser extent [10,11,12,13].

In monocots, promiscuity is not restricted to CYPs in rice diterpenoid metabolism. Two tandemly duplicated genes of the CYP71Z subfamily in maize, *CYP71Z16* and *CYP71Z18*, encode enzymes with partially redundant sequential activities on the precursors of both dolabralexins and kauralexins, including hydroxylations, epoxidations, and formation of carboxylic acids. A duplicate of the standard kaurene oxidase (CYP701) was also reported to be active on various intermediates of the grid-like kauralexin pathway [14,15].

In dicots, a similar scheme is observed in Lamiaceae, for example, in the medicinal Danshen sage (*Salvia miltiorrhiza*) where CYP76AH3, CYP76AK1, as well two members of the CYP71D subfamily (CYP71D373 and CYP71D375) were found to catalyze multiple reactions in the biosynthesis of the abietane *nor*-diterpenoid lactone bioactive group of compounds, tanshinones [16,17]. Of particular interest is CYP71D375, which was found to catalyze successive hydroxylation and heterocyclic D ring formation on three different backbone structures [17]. In *Coleus forskohlii*, another medicinal plant belonging to Lamiaceae, combinatorial expression of members of the CYP76AH subfamily revealed partially overlapping activities in the biosynthesis of forskolin from *13R*-manoyl-oxide, with CYP76AH11 showing a remarkable catalytic flexibility [18].

A broad substrate profile and catalytic versatility thus seem a common characteristic of the CYP enzymes contributing to the diterpenoid metabolism from conifers to angiosperms. This property evolved concomitantly with the evolution of terpene synthases, generating a diversity of olefinic backbones so as to produce a dynamic matrix of defense compounds. Yet, catalytic plasticity is also observed for CYP in other branches of the terpenoid metabolism, whether for biosynthesis or detoxification.

## 3. CYPs Involved in the Detoxification of Endogenous Toxins

Until recently, the concept that plant CYPs might contribute to the detoxification of noxious endogenous phytoalexins was just an interesting hypothesis. The major phytoalexins produced by Solanaceae are sesquiterpenoids like capsidiol, solavitevone or rishitin. In an effort to solve the pathway to rishitin, a member of the CYP76 family in potato, CYP76A2L, was spotted because it had orthologs in the tomato and tobacco genomes (potato, tomato and tobacco all produce solavitevone) but not in *Nicotiana benthamiana* (that does not produce solavitevone). CYP76A2L was thus tested for phytoalexin biosynthesis. The tests unexpectedly revealed that it is not involved in the solavitevone/rishitin biosynthetic pathway, but instead participates in the detoxification of a broad range of sesquiterpene phytoalexins via 13-hydroxylation of their side-chain. CYP76A2L detoxification capacity extends to sesquiterpenoid phytoalexins not present in potato, but prevalent in tobacco and pepper, such as capsidiol and capsidiol 3-acetate [19]. Other examples of promiscuous CYPs exapted for detoxification of endogenous toxins are thus likely to be discovered.

## 4. Beyond Diterpenoid or Endogenous Metabolism

In all cases reported above, the enzymes were tested for conversion of similar terpenoid structures related to the plant metabolic pathways under investigation, but the enzyme catalytic plasticity was not further explored. More striking examples of CYP enzyme promiscuity recently emerged in the context of smaller terpenoid and herbicide metabolism.

**Figure 2:**
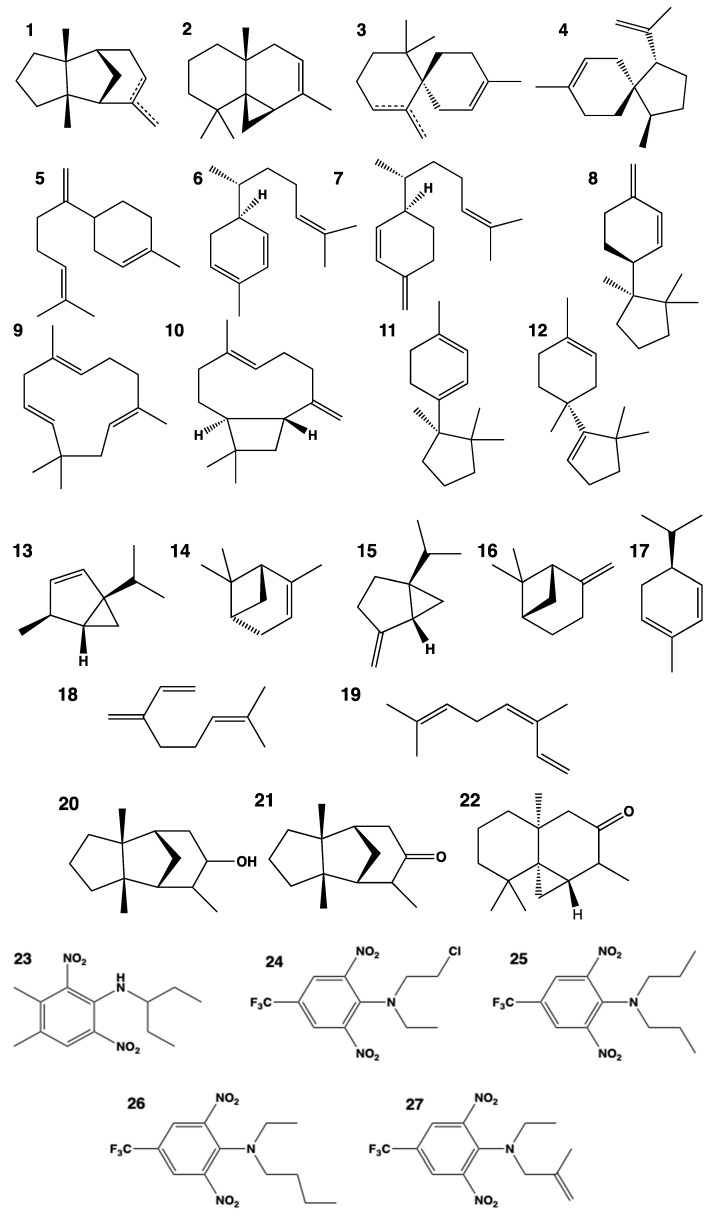
Natural and herbicide substrates of CYP706A3. Sesquiterpenes: **1**: (+)-α-barbatene and (+)-β-barbatene; **2**: (+)-thujopsene; **3**: (+)-α-chamigrene and (+)-β-chamigrene; **4**: β-acoradiene; **5**: (*E*)- γ-bisabolene; **6**: α-zingiberene; **7**: β-sesquiphellandrene; **8**: δ-cuprenene; **9**: α-humulene; 1**0**: (-)-(*E*)-β-caryophyllene; **11**: α-cuprenene; **12**: isobazzanene. Monoterpenes: **13**: β-thujene; **14**: α-pinene; **15**: (+)-sabinene; **16**: β-pinene; **17**: α-phellandrene; **18**: β-myrcene; **19**: β–*cis*-ocimene. Oxygenated sesquiterpenes: **20** (+)-6-OH-α-barbatene; **21**: (+)-6-oxo-barbatene; **22**: (+)-1-oxo-thujopsene. Herbicides: **23**: pendimethalin; **24**: fluchloralin; **25**: trifluralin; **26**: benefin; **27**: ethalfluralin.

*CYP706A3* is expressed upon the anthesis of Arabidopsis flowers. Unexpectedly, its suppression was found to result in the emission of a complex bouquet of mono- and sesquiterpenes. This observation led to the demonstration that, *in planta* and when expressed in yeast, CYP706A3 oxidizes more than twenty different mono- and sesquiterpenes to form oxygenated derivatives [20]. The primary products of CYP706A3-dependent oxidation can be further oxidized by the same enzyme, the oxygenated products being retained in plant tissues for the protection of reproductive organs against florivores and possibly also microbial parasites. CYP706A3 permissiveness was then further explored, testing the herbicide metabolism. This revealed its capacity to oxygenate and detoxify most of the active compounds of the family of dinitroanilines [21], thereby conferring a herbicide tolerance. A total of 29 different substrates are thus currently reported for this enzyme, all sharing a small size and high hydrophobicity (Figure 2). The capacity to metabolize dinitroanilines is conserved by CYP706s from Alaska cedar and eucalyptus [21].

Similarly, CYP76s from Arabidopsis have been reported to metabolize a range of monoterpenols such as geraniol, nerol, linalool citronellol, or lavandulol, with the precise spectrum of the monoterpenols metabolized varying between the family members [22,23]. They also convert some of their oxygenated derivatives. Like for CYP706A3, the suppression of CYP76C1 expressed in flowers upon anthesis led to a dramatic increase in floral volatile emission, in this case the emission of linalool. It also simultaneously prevented the emission of linalool oxides such as lilac aldehydes and lilac alcohols. CYP76C1 and several other Arabidopsis CYP76Cs have in addition been reported to detoxify the herbicides belonging to the class of phenylureas [22]. Other herbicides have been tested, but they were not converted. The capacity to metabolize monoterpenols, their derivatives and/or phenylurea seems to be widespread amongst CYP76s from different subfamilies in dicots [22,24].

**Figure 3:**
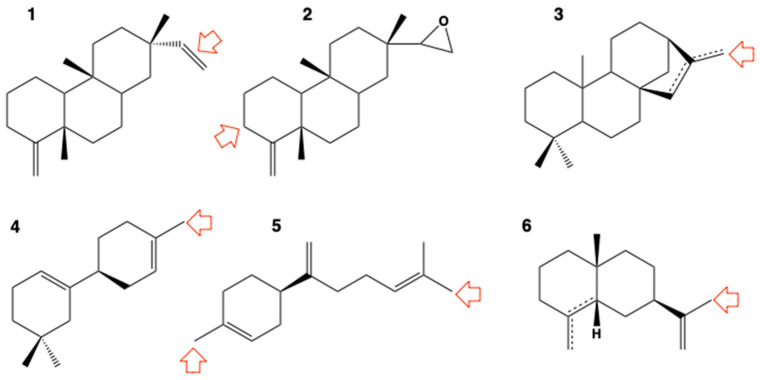
Substrates of the CYP71Z16/18/19 enzymes contributing to four terpenoid defense pathways in maize. The red arrows indicate the sites of oxidative attack: **1**: dolabriene and **2**: epoxydolabriene (precursors of dolabralexins); **3**: *ent*-kaurene or *ent*-isokaurene (precursors of kauralexins); **4**: β-macrocarpene and **5**: β-bisabolene (precusors of zealexins); **6**: β-selinene (precursor of costics acids). CYP71Z16 and CYP71Z18 were shown to oxidize **1**, **2**, **3**, 4 and **5**. CYP71Z19 was shown to convert **4**, **5** and **6**. β-bisabolene undergoes triple oxidation at the C1 or C15 positions.

Another striking example emerged from the exploration of the defense metabolism in monocots. Zealexins (non-volatile, acidic sesquiterpenoid derivatives of β-macrocarpene) form the largest class of terpenoid defense compounds in maize. A very comprehensive approach aiming to reveal their biosynthetic pathway showed that CYP71Z16, CYP71Z18 and CYP71Z19 catalyze multiple oxidations at one or several carbons on the β-macrocarpene and β-bisabolene scaffolds leading to the formation of zealexins. CYP71Z19 had additional activity on β-selinene for the formation of β-costic acid [25]. Maize CYP71Zs thus contribute to at least four different triterpenoid (kauralexins and dolabralexins) and sesquiterpenoid (zealexins and costic acids) pathways in maize (Figure 3). The presence of a functional and promiscuous ortholog in sorghum demonstrates that this enzyme’s multifunctionality evolved at least 12 million years ago [25]. In the same study, population mapping for zealexin ratios also revealed the involvement of a cluster of three CYP81A genes. Their expression in *N. benthamiana* demonstrated that CYP81A37 and CYP81A38 catalyze hydroxylation, desaturation and aromatization reactions on different intermediates to β-macrocarpene-derived zealexins.

**Figure 4:**
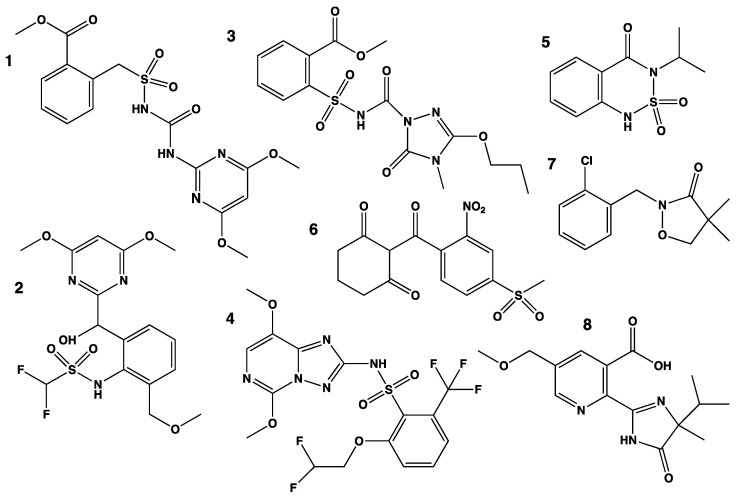
Representative members of the herbicide chemical groups metabolized by CYP81A12 and its paralogs of *Echinochloa phyllopogon*. Only one member of each chemical class is represented. Sulfonylurea are the best substrates, and among them bensulfuron-methyl is the most actively metabolized. The different CYP81A paralogs have different substrate preferences. The sites of the oxidative attacks leading to hydroxylation or demethylation have not been determined: **1**: bensulfuron-methyl (sulfonylurea); **2**: pyrimisulfan (pyrimidinyl(thio)benzoates); **3**: propoxycarbazone (sulfonylaminocarbonyl-triazolinones); **4**: penoxsulam (triazolopyrimidines); **5**: bentazone (benzothiadiazinones); **6**: mesotrione (triketones); **7**: clomazone; **8**: imazamox (imidazolinones).

The remarkable catalytic versatility of CYPs in the defense metabolism of monocots is likely to be related to the surprising promiscuity of CYP enzymes recently found responsible for herbicide polyresistance in grass weeds. Via genetic mapping and transcriptome analyses, members of the CYP81A subfamily were identified as most likely responsible for the herbicide cross-resistance observed in the grass weeds populations of *Lolium rigidum* and *Echinochloa phyllopogon* in Australia and California. That some of the members of the CYP81A subfamily indeed metabolize an unexpectedly broad set of herbicides was confirmed by heterologous expression in yeast and/or plants, demonstrating that, for example, CYP81A10v7 from *Lolium rigidum* [5] and CYP81A12/21/24 from *E. phyllopogon* conferred resistance and metabolized herbicides belonging to at least seven different chemical classes [6] (Figure 4). The presence of *E. phyllopogon* CYP81As orthologs in maize (belonging to the same subfamily of Poaceae: Panicoideae), with comparable promiscuity, and the characterization of a CYP81 with similar properties in *Lolium* (Pooideae of the BEP clade) indicate that this CYP81 metabolic plasticity did not recently evolve as an adaptation to herbicide treatments in weeds, but is an ancient acquisition of a common ancestor of Poaceae [26]. Fixation of these highly versatile CYP81As in grasses thus obviously required an ecological benefit. Their role in plant metabolism is so far not identified, but they are expected to further extend the complex system of chemical defense.

## 5. Promiscuity Revealed by Combinatorial Synthetic Biology Approaches

Synthetic biology has recently become a promising approach to discover novel CYP activities, in particular in the terpenoid pathways. Combinatorial expression of terpene synthases of one or several CYP enzymes has unveiled new CYP substrates and sometimes their capacity to form new-to-nature products. For example, a combinatorial co-expression platform used to elucidate the pathway of carnosic acid from miltiradiene in yeast revealed that CYP76AH24 and CYP76AK6 from sage (or CYP76AK8 from rosemary) accounted for at least six oxidation events required for the formation of the main labdane-type compounds found in these plants [27]. In a further experiment, the coexpression of a miltiradiene/abietadiene-generating module with different combinations of CYP76AK and CYP76AH enzymes led to the formation of 14 abietane diterpenoids, 8 of which had never been reported, disclosing additional diterpenoid substrates of these enzymes [28]. Another example was provided by the yeast co-expression of CYP720B1 with several combinations of diterpene synthases. This work demonstrated the capacity of CYP720B1 to process different labdane-type scaffolds and to generate additional products [29]. A similar combinatorial approach using terpene synthases from different organisms to generate 21 diterpene scaffolds, co-expressed in *E. coli* with CYP701s from rice and Arabidopsis, provided a quite extensive analysis of CYP701A3 and CYP701A6 promiscuity, enabling the formation and structural characterization of 12 unknown products [30].

## 6. Promiscuity Is Prominent in Blooming CYP Families

As the examples mentioned above clearly show, catalytic flexibility seems to be a characteristic commonly encountered in large CYP families showing a blooming pattern of gene duplications such as CYP71, CYP76, CYP81, or CYP706 [31]. These families are largely used to decorate the plethora of chemical scaffolds generated by terpene synthases, which are themselves versatile enzymes. This seems to make sense because enzyme promiscuity is expected to favor the emergence of novel selectable beneficial functions [32,33], especially in the context of pathways with a matrix-type structure such as the diterpenoid pathway. Enzyme promiscuity offers the possibility of simultaneous generation of a complex blend of defense compounds with a limited number of enzymes via natural combinatorial chemistry. This evolutionary outcome is most likely cost-effective to circumvent fast parasite or competitor adaptation. Genetic redundancy, coupled to partially overlapping substrate specificity, provides further resilience. Another common characteristic of these defense pathways is the strong inducibility of their gene expression by biotic or abiotic elicitors (including herbicide safeners) or transient expression upon flower anthesis, which is expected to minimize energy cost and deleterious effects on general plant metabolism.

## 7. Promiscuity Is also Present in Conserved CYP Families under Negative Selection

More surprisingly, promiscuity is also observed in families with a small number of genes. Such families are usually maintained under negative selection, because they control essential steps for the biosynthesis of hormones or key structural components such as lignin monomers. Such families are particularly interesting because they can demonstrate the transition from “single-copy control” to “multi-copy license” [34].

A good illustration of this transition is again found in the diterpenoid pathway. A single-copy *CYP701* gene is present in many plant species. This highly conserved CYP701 performs the triple monooxygenation of the C4 methyl of kaurene to form kaurenoic acid, the common precursor of the phytohormones gibberellins. Five CYP701 family members are, however, reported in rice but only one, CYP701A6, is required for the formation of gibberellic acid (GA). One of the duplicates, CYP701A8, also catalyzes the C3α-hydroxylation of *ent*-kaurene, but also the C3α-hydroxylation of two other labdane-type diterpenes sandaracopimaradiene and *ent*-cassadiene for the biosynthesis of the fungal phytoalexins oryzalexins and phytocassanes, respectively [10]. In plants, elicitation is required for *CYP701A8* expression and, consequently, no hydroxylated kaurene is detected. As already mentioned above, the substrate specificity of the single-copy CYP701A3 from Arabidopsis and of rice CYP701A6, both of which are required for the formation of GAs, was more systematically investigated using a combinatorial approach to test their capacity to oxidize 21 diterpene scaffolds [30]. This work unraveled a striking difference in their respective promiscuities. Rice CYP701A6 only catalyzed the triple monooxygenation at the C4α methyl of the closely related *ent*-kaurene, *ent*-isokaurene and *ent*-trachylobane. Conversely, CYP701A3 converted a broader set of diverse structures and either catalyzed the triple monoxygenation of the C4 methyl of scaffolds derived from *ent*-copalyldiphosphate (similar to *ent*-kaurene), or the hydroxylation of the C2 and C3 carbons on the opposite side of ring A of the other scaffolds. Interestingly, CYP701A6 evolved in Poaceae, which has produced a diversity of taxon-specific diterpenoid phytoalexins, while CYP701A3 is a representative of Brassicaceae that does not use the diterpenoid defense. Unfortunately, the CYP701A6 paralogs present in rice and devoted to defense metabolism were not included in this study. It nevertheless indicates that a tighter control of substrate selectivity in CYP from hormone metabolism, and probably at some stage negative selection, is required in the presence of competing pathways. The conversion of non-physiological substrates by CYP701A3 occurs with reduced catalytic efficiency. Yet the study suggests that even highly conserved CYPs might be prone to develop promiscuous activities in the absence of selection pressure. Such promiscuous activities provide an excellent background for seeding defense pathways. Accordingly, *CYP701* duplication and recruitment for defense pathways in Poaceae seems to be a repeated process [10].

Increased plasticity occurring in the CYP families dedicated to hormone metabolism does not necessarily lead to recruitment in defense pathways. All CYP711s that have been thus far functionally characterized, catalyze the triple oxygenation of carlactone into carlatonoic acid in the strigolactone biosynthetic pathway. CYP711 duplications have been observed in some plant taxa, for example in monocots. Their occurrence seems to lead to more promiscuous enzymes catalyzing additional reactions on strigolactone precursors, thus contributing to taxon-specific strigolactone structural and functional diversification [35,36].

Other interesting examples are in the phenylpropanoid pathway that conveys a huge flux of carbon for biosynthesis of lignin, in addition to antioxidants, UV screens and defense compounds. The plant phenolic pathway is very ancient and the CYPs in its most conserved sections evolved with plant terrestrialization.

**Figure 5:**
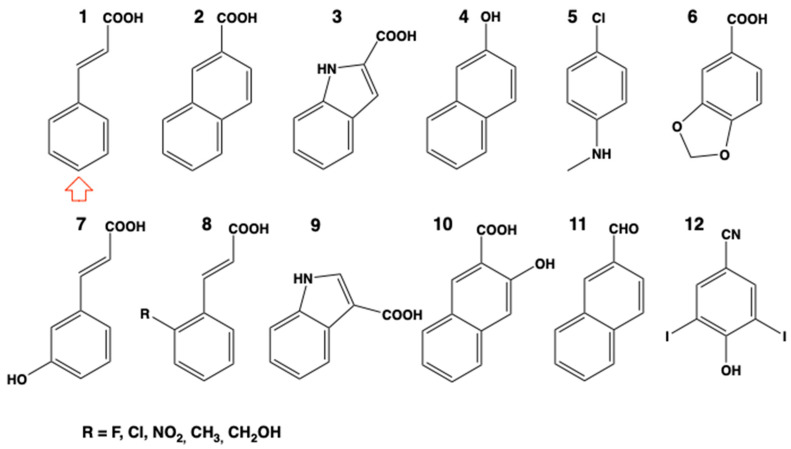
Substrate permissiveness and representative substrates and inhibitors of the highly conserved CYP73 in plant phenolic metabolism. Red arrow indicates the site of oxidative attack when determined: **1**: cinnamic acid (natural substrate); **2**: 2-naphtoic acid (hydroxylated with efficiency similar to cinnamic acid, used as a fluorescent reporter of CYP73 activity); **3**: indole-2-carboxylic acid; **4**: 2-naphtol; **5**: *p*-chloro-*N*-methylaniline; **6**: piperonylic acid (suicide substrate since attacked on methylene dioxo, used as a specific inhibitor of CYP73 *in planta*); **7**: *m*-coumaric acid; **8**: *o*-sustituted-cinnamic acids; **9**: indole-3-carboxylic acid; **10**: 1-hydroxy-2-naphtoic acid; **11**: 2-naphtaldehyde; **12**: ioxynil (herbicide not converted but ligand and competitive inhibitor (*K*_i_ = 2 μM)). Only *m*-coumaric acid, indole 3-carboxylic acid and piperonylic acid (extracted from bark of *Orotea pseudo-coto*) are natural plant compounds. Indole 3-carboxylic acid is reported to be present in high amounts in Arabidopsis [42] is converted with low efficiency. So far, no major variation in CYP73 substrate preference has been reported at any stage of the plant evolution, nor resulting from enzyme duplication.

CYP73 is involved in the core segment of the pathway and, as a cinnamate 4-hydroxylase, has to feed all branch pathways. This can require up to 30% of the carbon fixed by the plant. CYP73 is the only conserved CYP for which substrate permissiveness was explored using more systematic approaches. Several studies converged to indicate that only planar molecules of very similar shape and size can be significantly processed by the enzyme [37,38,39,40]. The best substrates were clearly cinnamic acid mimics: small, planar and aromatic compounds (Figure 5). Some were exploited to develop a fast fluorescent assay [38] and a specific enzyme inactivator [39]. Faint and selective herbicide metabolism and some strong xenobiotic ligands were also reported [40]. Such a substrate-specific, high flux-controlling CYP enzyme would be expected to be maintained as a single to low-copy gene in plant genomes. Indeed, CYP73 is present as a single copy in the genome of Arabidopsis, albeit duplicated in several ancestral taxa such as mosses or ferns. The functional reasons for such duplications have so far not been reported. Two well-fixed duplications are also conserved in Spermatophytes. The first occurred in the common ancestor of gymnosperms and angiosperms and the second in Monocots. All three copies maintained 4-hydroxylating activity on cinnamate with similar efficiencies [41]. As shown from the duplication of other conserved CYPs, this does not preclude acquisition of additional activity, but so far only modifications of membrane anchoring and orientation seem to be the novel functional property acquired by the earlier diverging CYP73 paralog fixed in some gymnosperms and most angiosperms [41].

The situation is completely different for the second enzyme of the pathway that controls the branchpoint to lignification. From its first characterization, CYP98 was shown to catalyze 3′-hydroxylation of the phenolic ring of both the very similar *p*-coumaroyl shikimate and *p*-coumaroyl-quinate with a very high efficiency [43]. The *p*-coumaroyl shikimate is the preferred substrate and seems to be used as an intermediate en route to the biosynthesis of biopolymers from the emergence of land plants to angiosperms [44]. Hydroxylation of *p*-coumaroyl-quinate is required for the biosynthesis of the UV screen and antioxidant chlorogenic acid and its derivatives. Contrary to CYP73, CYP98 gave rise to multiple taxon-specific gene duplications in angiosperms, in some cases for the enhanced production of chlorogenic acid, for example in coffee [45]. Quite early, functional screenings have been performed with orthologs and sometimes sets of paralogs, for instance the three functional copies present in wheat. These preliminary tests involving a limited number of substrates suggested that CYP98 catalytic plasticity varied with the species and paralogs, allowing low-rate conversion of *p*-coumaric acid and *p*-coumaroyl CoA, of 2-*O*-(4-coumaroyl)-*3*-(4-hydroxyphenyl)lactate and *p*-coumaroyl tyramine [46,47]. Non-conjugated natural phenolics with *p*-hydroxylated ring such as resveratrol were also found to be converted with low efficiency [47].

**Figure 6:**
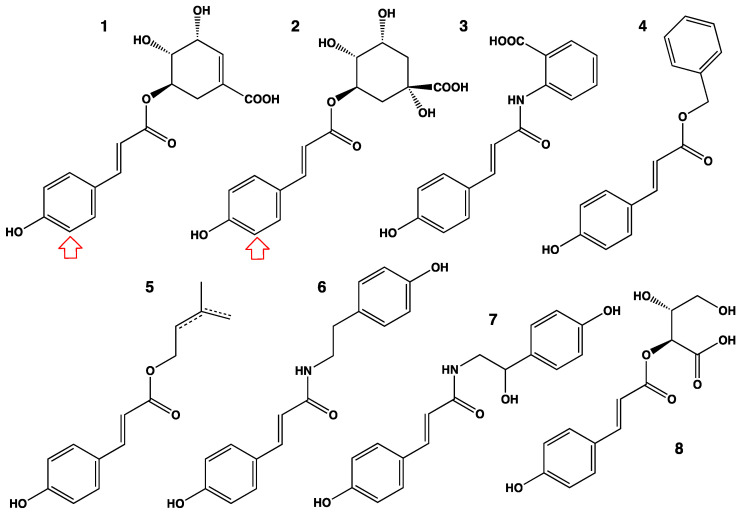

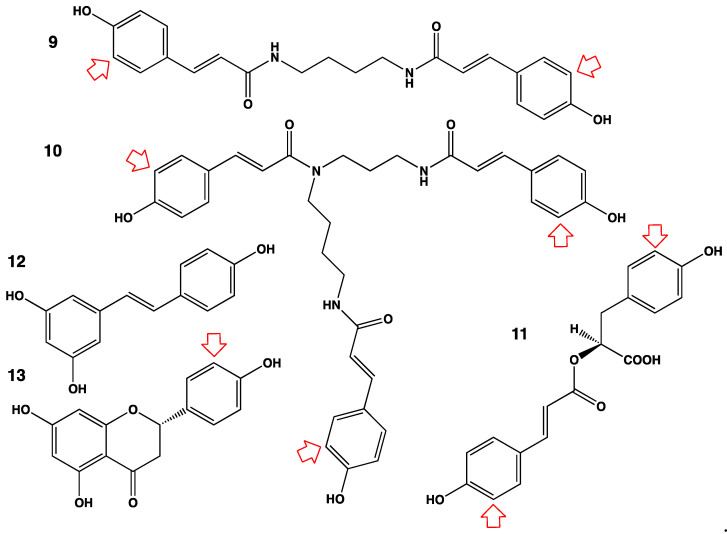
A representative sample of the confirmed substrates of CYP98 enzymes. Red arrow indicates the site of oxidative attack when determined. *p*-Coumaroyl-shikimate currently appears as the preferred substrate for CYP98s in Spermatophytes. *p*-Coumaroyl-anthranilate is the preferred substrate of CYP98s from Bryophytes and Lycophytes *in vitro* [49], yet *p*-coumaroyl-shikimate seems to be the most relevant substrate *in vivo* in moss [44]. Different enzymes (plant source or paralog) have different substrate preferences and all metabolize a range of conjugates with various efficiencies. Relaxation of substrate specificity and/or specialization occurs in some taxa, usually after gene duplication. Similar specialization can occur repeatedly with slightly different issues (e.g., [52]). In the vast majority of cases *p*-coumaroyl-shikimate activity is maintained in the specialized duplicates, at low to high levels. All substrates shown here have physiological relevance *in planta* as precursor of lignin, UV screen or defense compounds. **1**: 5-*O*-(*p*-coumaroyl)-shikimate (lignin precursor; highest turnover reported for an NADPH-dependent plant P450 enzyme); **2**: 5-*O*-(*p*-coumaroyl)-D-quinate (precursor of chlorogenic acid); **3**: *p*-coumaroyl-anthranilate; **4**: benzyl-*p*-coumarate; **5**: prenyl- or isoprenyl-*p*-coumarate; **6**: *p*-coumaroyl-tyramine; **7**: *p*-coumaroyl-octopamine; **8**: *p*-coumaroyl-4-threonate; **9**: *N,N’*-di-*p*-coumaroyl-putrescine (hydroxylated on both phenolic rings); **10**: *N*,*N*’,*N*”-tri-*p*-coumaroyl-spermidine (hydroxylated on the three phenolic rings); **11**: 2-*O*-(4-coumaroyl)-*3*-(4-hydroxyphenyl)lactic acid (precursor of rosmarinic acid; hydroxylated on both phenolic rings); **12**: *trans*-resveratrol; **13**: naringenin.

Evidence for even broader promiscuity emerged from the characterization of two tandemly duplicated paralogs (CYP98A8 and CYP98A9) generated via retroposition in Brassicales [48]. These CYPs clearly specialized for other activities, resulting in *meta*-ring hydroxylations of the three phenolic rings of tri-*p*-coumaroyl spermidine, to form a major component of the pollen coat/wall. It was then realized that this activity was already present at a very low level in the conserved ancestor representative CYP98A3. This triggered more systematic assays using an enlarged set of physiologically relevant phenolic esters and amides known to be present in plants, and a panel of CYP98 enzymes representative of land plant evolution. The latter included small subsets of taxon-specific duplicates likely to illustrate the functional evolution of paralogs after the duplication event. The activity screening unraveled quite extensive promiscuity, common to all CYP98 tested. Promiscuity was more pronounced in pine, and markedly enhanced in one of the duplicates when couples of paralogs were investigated [49] (Figure 6). Surprisingly, the best substrate for bryophytic enzymes *in vitro*, *p*-coumaroyl-anthranilate, was not the physiological substrate (*p*-coumaroyl-shikimate) that seems to be used by bryophytes *in planta* [44,50]. Several substrates are, however, likely to have been recruited by diverse plant species, for example by poplar to produce defense compounds [49]. The success of this recruitment relied on the presence of an appropriate conjugation enzyme and substrate pool.

The low CYP98 activities detected with non-conjugated substrates (Figure 6) were long considered as irrelevant *in vivo*. Surprisingly, upon functional validation of CYP98A9, it was recently observed that gain-of-function Arabidopsis mutants had a *transparent testa* phenotype (yellow seeds devoid of tannins). This indicated a flavonoid depletion in the seeds. CYP98A9 activity on flavonoids was then tested with the recombinant enzyme which was confirmed to catalyze the 3′-hydroxylation of naringenin. This activity was very low when measured *in vitro* and still had a clear impact on the plant flavonoid content, which is expected to affect seed viability [51].

These few examples illustrate several important points, some of which were well documented for other superfamilies of enzymes. The first is that the promiscuity of plant CYPs, even of the most essential ones, is largely overlooked. The second is that the best substrate *in vitro* is not necessarily the relevant substrate *in planta*. The third is that gene duplication favors drift to promiscuity of one of the paralogs and negative selection on the other one (as expected). The fourth is that a minor activity can become relevant in the plant if the enzyme is expressed at a sufficient level in a pertinent tissue. Moreover, even faint side-activities provide a lever (and are in fact most likely required) for positive selection and emergence of novel functions. 

Overall, the current data suggest that families of more promiscuous enzymes are more likely to bloom for recruitment to adaptive functions, especially if properly fed with diversified and structurally related substrates. This is particularly relevant in the terpenoid metabolism served by a large set of catalytically versatile and phylogenetically diversified terpene synthases. In the most ancestral and conserved CYP families, promiscuity is still encountered and may lead to novel activities or novel enzyme properties. Blooming seems nevertheless restrained by the necessity to maintain fluxes in essential pathways leading to negative selection on one of the duplicates.

## 8. Promiscuity from a CYP Family Point of View

The steadily increasing information on CYP function and the recent inventory of available data (online at: erda.dk/public/vgrid/PlantP450/, accessed on 15 November 2022) [31] currently offers an overview of the functional characteristics of each CYP family. This information reveals how the catalytic potential of the common ancestor of each CYP family most likely influenced each family’s functional evolution. Most CYP families are specialized for the metabolism of specific classes of metabolites. This is clearly the case for the small, conserved families. Yet, when the families expanded like in the CYP71 clan, a coincident expansion of the chemistries processed can be observed, with gene recruitment sometimes resulting in completely novel pathways.

For example, the largest CYP71 family is mainly dedicated to the terpenoid pathways (Table 1). Yet, CYP71 activities extend to other small volatile compounds such as oximes in the hydroxynitrile, cyanogenic glucoside and glucosinolate pathways. CYP71 activities on terpenoids and oximes are commonplace throughout angiosperms and catalyzed by enzymes belonging to different CYP71 subfamilies, hence it is unclear which activity predated in the common ancestor. Opportunistic gene duplicate recruitments have, in addition, occurred, shaping taxon-specific pathways such as monoterpene indole akaloids in Gentianales, indolic phytoalexins in Brassicaceae, benzoxazinones (DI(M)BOA) in Poaceae or furocoumarins in Apiaceae (for more see Table 1). Most frequent common properties of CYP71 substrates can be delineated as high hydrophobicity and quite often small size (all the smallest plant CYP substrates are converted by CYP71s), but with some significant divergences from this scheme. Another expanded family, CYP76, is also most often acting in the terpenoid metabolism, processing monoterpenes, sesquiterpenes, diterpenes, and other compounds with isoprenoid side-chains (e.g., demethylsuberosin or geranylquinone). Most of the CYP76 substrates have at least one alcohol substituent (e.g., monoterpenols). CYP76s frequently catalyze multiple oxidations at the same carbon that can result in ring formation. In this case, the most surprising and clearly opportunistic recruitment occurred for the hydroxylations of tyrosine and DOPA in the betalain pathway. The CYP71 and CYP76 examples seem compatible with progressive shifts in substrate specificity/plasticity. Their ancestor reconstruction could help to track ancestral activity of the founding enzyme(s) and to understand the observed functional divergences.

The CYP81/82 families are more intriguing. As families, CYP81 and CYP82 stem from a common ancestor, and have in common the participation in an unusual number of different plant pathways and diverse reactions with no clear preference for a class of substrates (Table 1). Together with the recent demonstration that CYP81s from *E. phyllopogon* metabolize seven different classes of herbicides with different physicochemical properties [26], this would be in line with an innate promiscuity not encountered in other CYP clades. It suggests a remarkable pliability of the CYP81/82 ancestor and calls into question the level of (and reason for) promiscuity maintained throughout the evolution of this clade.

All families outside of the CYP71 clan are clearly more specialized. The most diversified is CYP72, which besides inactivation of brassinosteroid hormones (only in Brassicales) and different hydroxylations of gibberellins, has been mainly recruited for the biosynthesis of triterpenoid defense, and to a lesser extent of diterpenoids, iridoids and terpene indole alkaloids.

## 9. Structural Determinants of CYP Promiscuity

The structural determinants of CYP promiscuity have been illustrated elsewhere [33]. The size of the active site and the physicochemical properties of the amino acids lining it are of course important determinants. However, the CYP active site is not readily accessible from the solvent, since located in the protein core. This implies an important control of the access channel(s), their number, size, hydrophilicity, plasticity (see e.g., [21]). The overall protein flexibility and possible presence of a hinge region, allowing opening of the active site, might also play an important role in substrate accessibility as suggested in the case of the plant CYP98A8/9 [48], and well demonstrated by structural elucidation of the ligand-bound bacterial CYP from *Streptomyces antibioticus* involved in the final steps of the biosynthesis of the antibiotic oleandomycin [53].

## 10. Exploring CYP Promiscuity

It would of course be appealing to obtain a more comprehensive and broader overview of the plant CYP promiscuity and catalytic potential. Such a global picture would be critical to understand the functional evolution of the superfamily and to anticipate potential metabolic interferences due to multiple or side functions in the plant. It would also provide important support for metabolic engineering and for the prediction and control of herbicide/pesticide resistance while taking into account the physiological consequences of pesticide treatment for plant development and defense. A first challenge for an effective functional screening would be to collect a representative set of compounds in amounts sufficient to screen a large number of enzymes. The second one would be to express the enzymes in a functional form and amounts sufficient for a large number of assays. Last but not least, no generic approach seems to be viable for P450 activity detection. Different assays, based on NADPH or oxygen consumption have been designed, leading to some hits [54,55], but they have proved unsuitable for providing a reliable global picture. Many ligands have access to the CYP active site and, instead of being oxygenated, trigger abortive catalytic cycles, only leading to the release of activated oxygen. Poor substrates or just ligands with nonoptimal positioning in the active site also lead to reaction uncoupling. Generic approaches thus suffer from very high proportions of false positives [56]. In addition, measurement of NADPH or oxygen consumption by the reaction requires the use of microsome or membrane preparations. For all these reasons, the most realistic approach with large enzyme collections seems to be combinatorial screening using whole recombinant microorganisms and product detection via LC- or GC-MS, which is still a daunting task.

## 11. Outlook

Plant cytochrome P450 promiscuity has been clearly overlooked. It is now obvious that the catalytic plasticity of P450 enzymes plays a critical role in the process of adaptation and evolution of novel metabolic pathways. This plasticity is also likely to influence the dynamics of the plant CYP family evolution. Although a systematic screening of plant CYP functions does not yet seem like an achievable goal, a more thorough metabolic profiling of the CYP loss or gain function of plants might reveal unexpected catalytic properties in the future. Random enzyme associations in metabolic engineering processes could be another source of useful information to guide further investigations.

## Figures and Tables

**Table 1:** Catalytic activities reported for the most diversified (blooming) plant CYP families. Frequency refers to the number of enzymes investigated and shown to metabolize a class of compounds. N.B. the table is exclusively based on the currently available erda.dk/public/vgrid/PlantP450/ database that is focused on plant physiological activities, but not exhaustive regarding metabolism of xenobiotics. The database will be updated in the coming months in this respect. References of the publications reporting activities of the members of each family can be found in the database.

P450 Family	Reported Substrates	Frequency	Comments
CYP71	aminoacid-derived oximes monoterpene(oid)s sesquiterpene(oid)s diterpene(oid)s triterpene(oid)s monoterpene indole alkaloids benzoxazinones (furo)coumarins indolic derivatives (besides oxime) lignans flavonoids tryptamine myristicin (phenylpropenes) benzoquinoline alkaloids benzoquinones jasmone (oxylipins) herbicides (phenylurea) *p*-chloro-*N*-methylaniline	12 14 29 20 10 14 12 * 8 3 2 2 1 1 1 1 1 1 1	cyanogenogenic glucosides, hydroxynitriles rarely monoterpenols in Gentianales in Poaceae (DI(M)BOA) in Apiaceae in Brassicaceae podophyllotoxin/etoposide serotonin perilla aroma colchicin sorgoleone/allelopathy pyrethrins CYP71A1 (first plant CYP activity reported)
CYP72	brassinosteroids gibberellins triterpenes iridoids herbicides (ALS inhibitors)	1 11 30 7 1	inactivation, CYP72C1 several GAs, often dual activities, CYP72As in monocots and dicots in monocots and dicots, CYP72As, convergent evolution bispyrac-methyl, bensulfuron-methyl
CYP76	monoterpene(ol)s/iridoids sesquiterpenes diterpenes tyrosine and DOPA geranylquinones demethylsuberosin herbicides (phenylurea only)	13 12 21 4 3 1 4	monoterpenols essentially, in several subfamilies Poaceae (rice), Lamiales betalain pathway on prenyl chain, shikonin on prenyl chain, furocoumarins
CYP81	fatty acids isoflavones lignans xanthones sesquiterpenoids diterpenoids triterpenoids indole glucosinolates herbicides (several classes)	1 3 2 3 3 2 4 1 9	in chain hydroxylation methylene dioxo formation phenol coupling orthologs in monocots, CYP81As
CYP82	flavonoids ** coumarins lignans indole 3-carboxy nitrile nerolidol/geranylinalool sesquiterpenes triterpenes terpene indole alkaloids nicotine isoquinoline alkaloids tropane alkaloids naphtoquinones jasmolone (oxylipins)	12 6 1 2 3 1 1 2 4 5 1 4 1	in several subfamiles in several subfamilies Arabidopsis indolic defense cleavage, homoterpene biosynthesis dealkylation -> *nor*-nicotine desaturation -> pyrethrins

* Three sets of orthologs; ** nine paralogs in same genus.

## Data Availability

This a review based on already published and publicly available data.
